# MCRS1 associates with cytoplasmic dynein and mediates pericentrosomal material recruitment

**DOI:** 10.1038/srep27284

**Published:** 2016-06-06

**Authors:** Si-Hyung Lee, Mi-Sun Lee, Tae-Ik Choi, Hyowon Hong, Jun-Young Seo, Cheol-Hee Kim, Joon Kim

**Affiliations:** 1 Graduate School of Medical Science and Engineering, KAIST, Daejeon 34141, Korea; 2 Department of Biology, Chungnam National University, Daejeon 34134, Korea; 3 Severance Biomedical Science Institute, Brain Korea 21 PLUS Project for Medical Science, Yonsei University College of Medicine, Seoul 03722, Korea

## Abstract

MCRS1 is involved in multiple cellular activities, including mitotic spindle assembly, mTOR signaling and tumorigenesis. Although MCRS1 has been reported to bind to the dynein regulator NDE1, a functional interaction between MCRS1 and cytoplasmic dynein remains unaddressed. Here, we demonstrate that MCRS1 is required for dynein-dependent cargo transport to the centrosome and also plays a role in primary cilium formation. MCRS1 localized to centriolar satellites. Knockdown of MCRS1 resulted in a dispersion of centriolar satellites whose establishment depends on cytoplasmic dynein. By contrast, NDE1 was not necessary for the proper distribution of centriolar satellites, indicating a functional distinction between MCRS1 and NDE1. Unlike NDE1, MCRS1 played a positive role for the initiation of ciliogenesis, possibly through its interaction with TTBK2. Zebrafish with homozygous *mcrs1* mutants exhibited a reduction in the size of the brain and the eye due to excessive apoptosis. In addition, *mcrs1* mutants failed to develop distinct layers in the retina, and showed a defect in melatonin-induced aggregation of melanosomes in melanophores. These phenotypes are reminiscent of zebrafish dynein mutants. Reduced ciliogenesis was also apparent in the olfactory placode of *mcrs1* mutants. Collectively, our findings identify MCRS1 as a dynein-interacting protein critical for centriolar satellite formation and ciliogenesis.

Microspherule protein 1 (MCRS1) and its isoform 58-kDa microspherule protein (MSP58) are localized to electron dense bodies within the nucleoli, designated as microspherules^1^. MCRS1 plays divergent roles in the regulation of gene expression and cell proliferation by interacting with various proteins including proliferation-related nucleolar protein NOP2, transcriptional corepression Daxx, and telomerase-inhibitory protein LPTS/PinX1^2^,^3^. Overexpression of MCRS1 induces anchorage-independent growth of avian fibroblasts, and also promotes metastatic behavior of lung cancer cells^4^,^5^. Contrarily, depletion of MCRS1 reduces the growth of glioma and colorectal cancer cells^6^,^7^. The tumor suppressor PTEN was shown to bind to MCRS1 and inhibit its oncogenic activity^8^. In addition, a recent study demonstrated that MCRS1 is involved in the activation of the mTOR pathway in response to amino acids. MCRS1 appears to provide a link between mTOR complex 1 and its positive regulator Rheb^9^.

Moreover, MCRS1 has been implicated in microtubule-dependent cellular processes. The assembly of a bipolar spindle in mitotic cells requires MCRS1 function. MCRS1 localizes to the minus ends of both chromosomal and kinetochore microtubules, and MCRS1 protects them from depolymerization in a RanGTP-dependent manner^10^. In addition, MSP58 isoform of MCRS1 interacts with fragile X mental retardation protein (FMRP/FMR1), which is an RNA binding protein that is involved in the regulation of mRNA transport along microtubules for localized protein synthesis. Although precise mechanism is not clear, MSP58 appears to escort FMRP-bound mRNPs from the nucleus to the somato-dendritic compartment of hippocampal neurons^11^. Previously, MCRS1 was identified as an interaction partner of NDE1, a regulator of cytoplasmic dyneins moving along microtubules. MCRS1 forms a complex with NDE1 and Su48/ZNF365, and they are co-localized to the centrosome^12^. However, potential roles of MCRS1 in dynein or centrosomal function remain unaddressed.

Cytoplasmic dynein is a minus-end-directed microtubule motor which performs critical functions in a wide range of cellular activities including organelle positioning, mitosis and cell migration^13^,^14^,^15^. Cytoplasmic dynein assembles around a force-generating heavy chain dimer, which serves as a platform for the binding of intermediate chain, light-intermediate chain, and three types of light chain (LC8, TCTEX and Roadblock)^16^. The core subunits of cytoplasmic dynein interact with several adaptor proteins, which modulate the mechanical behavior of the motor and also support their cargo-binding activities^16^. NDE1 is one of the ubiquitous dynein regulators which promote the transport of high-load cargoes^17^. NDE1 has been shown to assist dynein-dependent steps in mitosis and cell migration^18^,^19^. NDE1 binds to both intermediate chains of the cytoplasmic dynein 1 complex and the LC8 isoform of light chains^18^,^20^. Interestingly, recent studies have demonstrated that NDE1 interacts with LC8 in the basal body area to suppress the elongation of primary cilia^21^. NDE1 appears to coordinate ciliary length with cell cycle progression [20].

Centriolar satellites are electron-dense, spherical granules of 70–100 nm diameters that are scattered around the centrosome^22^,^23^. The granules are associated with microtubules originating from the centrosome, and their motility and pericentrosomal accumulation depends on cytoplasmic dynein 1^22^. There is accumulating evidence that centriolar satellites play roles in the recruitment and assembly of centrosomal proteins. Pericentriolar material 1 (PCM1) is a major component of centriolar satellites. PCM1 depletion was shown to reduce centrosomal localization of a subset of centrosomal proteins such as centrin, pericentrin and ninein^24^. Moreover, a recent study found that excessive formation of centriolar satellites promotes centrosome amplification after DNA damage^25^. Although disruption of centriolar satellites by PCM1 knockdown does not interfere with *de novo* centriole assembly and ciliogenesis in cultured tracheal epithelial cells^26^, centriolar satellites have been repeatedly associated with proteins implicated in cilia-associated genetic disorders called the ciliopathies. Ciliopathy proteins CEP290, BBS4 and OFD1 are detected in PCM1-containing centriolar satellites, and the ciliopathy proteins are mutually dependent for their centriolar satellite localization^27^,^28^,^29^. Centriolar satellites may function as assembly points for the ciliopathy proteins, allowing fine-tuning of the composition of ciliary components^24^,^27^,^28^.

Dynamic yet consistent distribution patterns of centriolar satellites during the progression of cell cycle suggest that the interaction between centriolar satellites and motor proteins are tightly controlled. However, it is largely unclear how specific dynein pools are assigned for centriolar satellites and how their activities are regulated in accordance with the assembly of centrosomal or ciliopathy protein complexes. Here, we demonstrate that MCRS1 interacts with the dynein intermediate chain DYNC1I1, and plays an essential role in both the establishment of centriolar satellites and the initiation of primary cilium assembly.

## Results

### MCRS1 binds to cytoplasmic dynein and contributes to the establishment of centriolar satellites.

**Figure 1:**
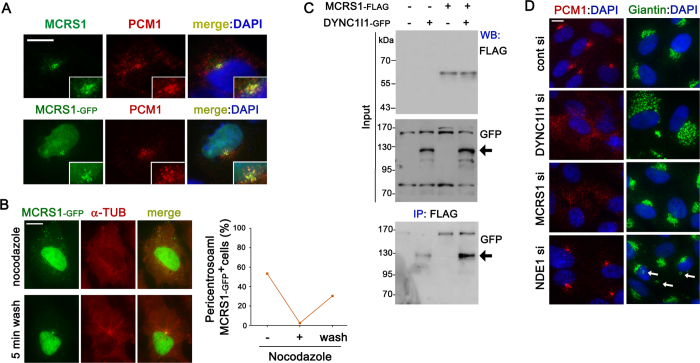
MCRS1 is required for proper distribution of centriolar satellites. (**A**) Immunofluorescence images showing the distribution of endogenous MCRS1 and the centriolar satellite marker PCM1 (upper panels). RPE1 cells transiently expressing EGFP-tagged MCRS1 were stained with PCM1 antibody (lower panels). (**B**) RPE1 cells transiently expressing EGFP-tagged MCRS1 were treated with nocodazole for 1 hr, and then stained with α-Tubulin antibody with or without nocodazole washout (5 min wash). The graph shows quantification of cells exhibiting pericentrosomal accumulation of MCRS1-GFP-positive granules: (-) without nocodazole treatment, (+) after 1 hr nocodazole treatment, and (wash) after 1 hr nocodazole treatment followed by 5 min washout. (**C**) HEK293T cells were transfected with the indicated plasmids for 16 hr, and then cell lysates were immunoprecipitated with anti-FLAG antibody conjugated with agarose beads. The resulting precipitates and input lysates were immunoblotted with the indicated antibodies. Arrow indicates bands at the molecular weight of DYNC1l1-GFP. (**D**) Immunofluorescence images showing the distribution of PCM1-containing granules and the Golgi complex after transfection of the indicated siRNAs for 3 days. Scale bars represent 10 μm.

We examined the subcellular localization of MCRS1 in telomerase-immortalized human retinal pigmented epithelial (RPE1) cells. We observed that anti-MCRS1 antibody stains scattered particles around the centrosome which colocalized with the centriolar satellite marker PCM1. An immunofluorescence analysis of RPE1 cells transfected with MCRS1-GFP further confirmed the localization of MCRS1 to centriolar satellites (Fig. 1A). Centriolar satellites are known to be maintained by microtubule-based motility mediated by cytoplasmic dynein^30^. To determine if microtubules are required for pericentrosomal recruitment of MCRS1, we examined the effect of nocodazole on the distribution of MCRS1-GFP. As shown in Fig. 1B, disruption of microtubules resulted in a dispersion of MCRS1-GFP throughout the cytoplasm. Pericentrosomal accumulation of MCRS1-GFP was recovered in 5 min after nocodazole-washout which allowed microtubule regrowth from the centrosome (Fig. 1B). To further visualize the motility of MCRS1-GFP+ particles, we performed time-lapse imaging of live RPE1 cells expressing both MCRS1-GFP and DsRed-conjugated Centrin2, which is recruited to the centrosome through centriolar satellites^24^. A fraction of pericentrosomal particles double positive for MCRS1-GFP and Centrin-DsRed exhibited directional motility (Supplementary Fig. S1). Interestingly, MCRS1-GFP consistently moved ahead of Centrin-DsRed although the two proteins colocalized to the same particles before and after the movement. This observation suggests that MCRS1 and Centrin2 do not bind directly, and MCRS1 is more closely linked to motor proteins than Centrin2.

Cytoplasmic dynein mediates the movement of centriolar satellites^30^. Thus, we examined the association of MCRS1 with the cytoplasmic dynein intermediate chain DYNC1I1, which is a central binding platform for multiple dynein adaptors including NDE1. Our co-immunoprecipitation analysis detected a physical interaction between MCRS1 and DYNC1I1 (Fig. 1C). In addition, exogenous MCRS1 colocalized with the DYNC1I1/NDE1 complex in the cytoplasm and the nucleus, and the complex also recruited MCRS1 to lamellipodia (Supplementary Fig. S1). To test if MCRS1 might modulate the interaction between DYNC1I1 and NDE1, we performed co-immunoprecipitation of DYNC1I1-GFP and NDE1-MYC in the presence or absence of co-transfected MCRS1. As shown in Supplementary Fig. S1, co-expression of MCRS1-FLAG inhibited the interaction between DYNC1l1-GFP and NDE1-MYC (Supplementary Fig. S1). This raises the possibility that MCRS1 and NDE1 may form functionally distinct complexes with cytoplasmic dynein.

To determine whether MCRS1 is a passive cargo or a mediator of the movement of centriolar satellites, we examined PCM1 distribution in RPE1 cells transfected with MCRS1 siRNAs. As shown in Fig. 1D, particulate PCM1 staining was accumulated in the pericentrosomal area in cells transfected with control (non-targeting) siRNA. By contrast, PCM1-containing particles were dispersed throughout the cytoplasm in cells depleted of either DYNC1I1 or MCRS1, indicating that both dynein and MCRS1 are required for proper distribution of centriolar satellites (Fig. 1D). Another MCRS1 siRNA exerted similar effect on PCM1 distribution (Supplementary Fig. S2), and MCRS1 depletion was confirmed by western blot and immunofluorescence analyses (Supplementary Fig. S2). Remarkably, depletion of NDE1 did not affect the distribution of PCM1-containing centriolar satellites (Fig. 1D). By contrast, Golgi positioning, which depends on dynein function, was not affected by depletion of MCRS1, while NDE1 knockdown increased fragmented Golgi membranes (Fig. 1D). Although MCRS1 was originally identified as a NDE1 binding protein, our observations suggest that the role of MCRS1 in the formation of centriolar satellites is independent of NDE1 activity. Loss of centriolar satellites has been shown to reduce the recruitment of a subset of centrosomal proteins including Pericentrin^24^. In accordance with previous reports, defects in centriolar satellites caused by MCRS1 knockdown decreased Pericentrin recruitment to the centrosome, while centrosomal γ-Tubulin levels were unaffected (Supplementary Fig. S2). These results indicate that MCRS1 plays an active role in the establishment and function of centriolar satellites.

### N-terminal truncated MCRS1 exerts a dominant-negative effect on dynein function

**Figure 2:**
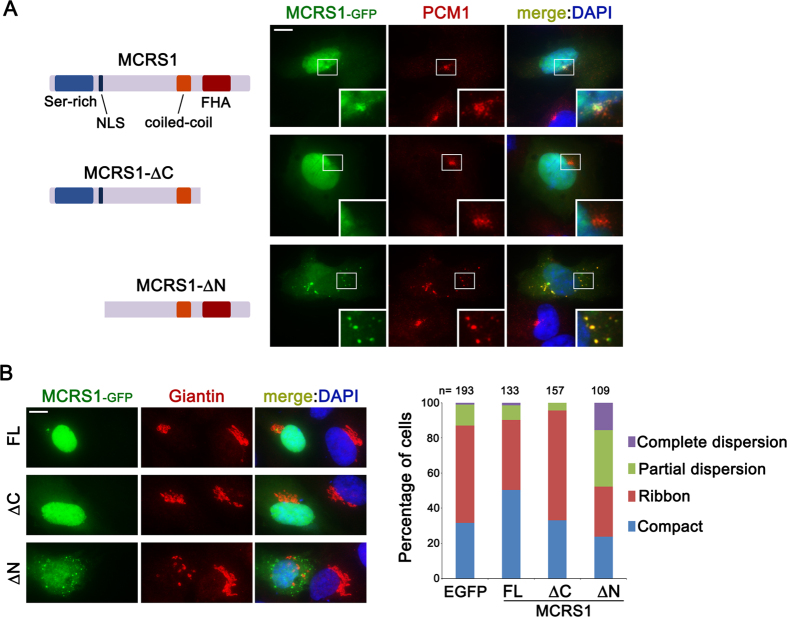
N-terminal truncated MCRS1 impairs both centriolar satellite distribution and Golgi organization. (**A**) Schematic representation of domain structures of MCRS1, and subcellular localization of truncated forms of MCRS1. RPE1 cells transiently expressing the indicated constructs were stained with anti-PCM1 antibody. Insets are magnified view of the centrosomal area. (**B**) RPE1 cells expressing the indicated constructs were stained with anti-Giantin antibody. Quantification of the influence of the indicated constructs on Golgi organization visualized as in B. Transfected cells were classified into four groups based on the distribution of the Golgi apparatus. Scale bars represent 10 μm.

MCRS1 has three predicted protein domains: serine-rich domain, coiled-coil domain and forkhead-associated (FHA) domain (Fig. 2A). In addition, MCRS1 contains a bipartite nuclear localization signal (NLS) near the serine-rich domain. We generated two truncated MCRS1 constructs, MCRS1-∆C and MCRS1-∆N. MCRS1-∆C lacks the FHA domain, which is known to mediate both the interaction with NDE1 and centrosomal localization^12^. As expected, MCRS1-∆C localized to the nucleus, but was not detected from the centrosome and centriolar satellites (Fig. 2A). By contrast, MCRS1-∆N, which lacks both the serine-rich domain and NSL, formed cytoplasmic aggregates much larger than centriolar satellites, and failed to accumulate in the nucleus. Notably, the aggregates recruited PCM1, and also disrupted normal distribution of PCM1-containing centriolar satellites. To test if MCRS1-∆N exerted a dominant-negative effect on dynein function, we examined potential changes in Golgi organization in cells transfected with truncated MCRS1 constructs. The Golgi was observed as compact stacks or ribbon-like shape around the nucleus in cells expressing control (EGFP) or MCRS1-∆C (Fig. 2B). The fraction of cells showing partially or completely fragmented Golgi membranes was increased in response to MCRS1-∆N expression (Fig. 2B). It is possible that MCRS1-∆N may inhibit the activity of dynein complexes or sequester them to PCM1-containing aggregates. Although MCRS1 knockdown did not disrupt Golgi positioning (Fig. 1D), overexpression of wild-type MCRS1 caused an increase in the number of cells exhibiting compact Golgi stacks (Fig. 2B). Together, these results suggest that MCRS1 exerts an effect on the activity of cytoplasmic dynein.

### MCRS1 is involved in ciliogenesis and TTBK2 recruitment to the mother centriole

**Figure 3:**
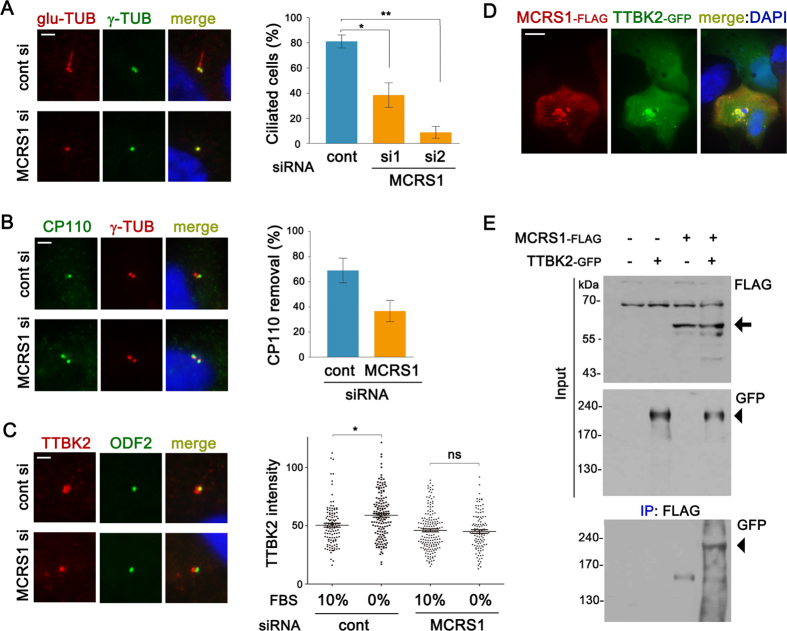
MCRS1 is required for ciliogenesis and TTBK2 recruitment to the mother centriole. (**A**) RPE1 cells were transfected with siRNAs for 24 hr, and then serum-starved for 2 days. Cilia were stained with anti-polyglutamylated Tubulin antibody. The graph shows quantification of ciliated cells. Error bars represent SEM (n=3 independent experiments; *P < 0.05 and **P < 0.01, t test). (**B**) Immunofluorescence analysis of CP110 cap removal from the mother centriole. Cells were transfected with siRNAs for 48 hr, and then serum-starved for 24 hr. The graph shows quantification of cells with single CP110 dot in the centrosome. Error bars represent SEM (n=3 independent experiments). (**C**) Immunofluorescence analysis of TTBK2 recruitment to the mother centriole. Cells were transfected with siRNAs for 56 hr, and then serum-starved for 16 hr. The scatter plot shows quantification of immunofluorescence intensities of TTBK2 at the centrosomal area. Error bars represent SEM (more than 100 cells were examined for each group; *P < 0.05, t test). (**D**) Immunofluorescence images of a RPE1 cell expressing MCRS1-FLAG and TTBK2-GFP. (E) HEK293T cells were transfected with the indicated plasmids for 16 hr. Cell lysates were immunoprecipitated with anti-FLAG antibody conjugated with agarose beads. The resulting precipitates and input lysates were immunoblotted with the indicated antibodies. Arrow and arrowhead indicate bands at the molecular weight of MCRS1-FLAG and TTBK2-GFP, respectively. Scale bars represent 10 μm (**A** and **D**) and 2 μm (**B** and **C**).

The frequent association between ciliopathy proteins and centriolar satellites suggests that efficient ciliogenesis or ciliary function depends on centriolar satellites. To test the involvement of MCRS1 in ciliogenesis, we examined the impact of MCRS1 knockdown on serum starvation-induced ciliogenesis in RPE1 cells. As shown in Fig. 3A, cells transfected with MCRS1 siRNAs showed an impairment of the assembly of primary cilia. Removal of CP110 cap from the mother centriole has been shown to be one of the earliest steps in ciliogenesis^31^. Compared with controls, the number of cells maintaining CP110 cap from both the mother and the daughter centrioles was increased by MCRS1 siRNA transfection (Fig. 3B). TTBK2 has been identified as a key molecule in the process of CP110 cap removal, and serum starvation promotes the recruitment of TTBK2 to the mother centriole^32^. Thus, we next tested if MCRS1 is required for the recruitment of TTBK2 to the mother centriole at the onset of ciliogenesis. Remarkably, serum starvation-induced centriolar TTBK2 recruitment was not observed in cells transfected with MCRS1 siRNA (Fig. 3C). To understand the mechanism of impaired TTBK2 recruitment, we examined potential physical interaction between MCRS1 and TTBK2. In cells expressing both MCRS1-FLAG and TTBK2-GFP, MCRS1-FLAG failed to accumulate in the nucleus, whereas large cytoplasmic aggregates containing both MCRS1-FLAG and TTBK2-GFP appeared (Fig. 3D). Moreover, the interaction between MCRS1 and TTBK2 was confirmed by a co-immunoprecipitation analysis (Fig. 3E). These findings suggest that MCRS1 mediates centriolar recruitment of TTBK2 through physical interaction with TTBK2.

We next examined the effect of TTBK2 on MCRS1-NDE1 interaction. Interestingly, overexpression of TTBK2 exerted a positive effect on the physical interaction between MCRS1 and NDE1 (Supplementary Fig. S3). Therefore, it is likely that there is a close relationship between MCRS1, NDE1 and TTBK2. Previously it has been suggested that the interaction between MCRS1 and NDE1 is regulated by phosphorylation on NDE1^12^. Thus, we speculate that NDE1 could be a target of TTBK2-mediated phosphorylation.

It has been shown that the ciliopathy protein CEP290 localizes to centriolar satellites, and plays a role for the assembly of primary cilia^27^,^33^. Similar to PCM1, CEP290-containing particles that normally cluster around the centrosome largely disappeared after MCRS1 knockdown (Supplementary Fig. S4). Thus, ciliogenesis defects observed in MCRS1 depleted cells might be ascribed to inefficient pericentrosomal recruitment of multiple cilia-associated proteins. As shown in Supplementary Fig. S4, a pool of MCRS1-GFP was detected inside the cilium, while the majority of MCRS1 still localized to the nucleus. Centriolar satellite localization of MCRS1-GFP became less prominent in ciliated cells. This observation may hint at a role for MCRS1 in the maintenance or functions of primary cilia.

### Zebrafish mcrs1 mutants exhibited excessive neuronal death, impaired melanosome recruitment and olfactory ciliogenesis defect

**Figure 4:**
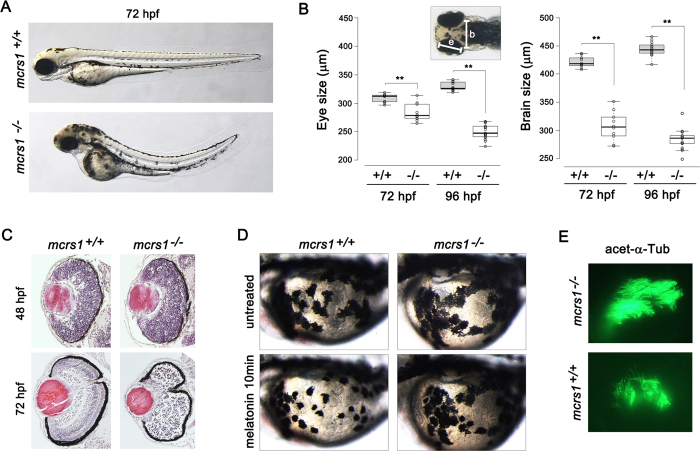
TALEN-mediated knockout of mcrs1 in zebrafish. (**A**) Homozygous mcrs1 mutant zebrafish exhibited a curved body axis at 72 hpf. (**B**) Measurement of the size of the eye and the brain (n>10 for each genotype; **P < 0.01, t test). (**C**) Disruption of retinal lamination in mcrs1 mutant zebrafish at 72 hpf. (**D**) Melatonin-induced aggregation of melanosome was delayed in mcrs1 mutant zebrafish. (E) Cilia in the olfactory placode were visualized by staining with anti-acetylated tubulin antibody.

Next, we investigated the *in vivo* function of MCRS1. The zebrafish mcrs1 protein reveals a high level of similarity in the conserved domain to its human counterpart (Supplementary Fig. S5). Using whole-mount *in situ* hybridization, we analyzed the spatiotemporal expression of *mcrs1* during zebrafish embryonic development. By 30 hour post-fertilization (hpf), widespread expression of *mcris1* was evident within the brain, the spinal cord and the pronephros (Supplementary Fig. S5). *mcrs1* transcripts were mainly observed in the developing brain and eyes at 48 and 60 hpf (Supplementary Fig. S5). To generate zebrafish *mcrs1* knockout mutants, we used transcription-activator-like effector nuclease (TALEN) technology. We injected a pair of TALEN mRNAs targeting the exon 4 of *mcrs1* into 1-cell stage zebrafish embryos. We performed T7 endonuclease I (T7E1) assay, and identified a germline mutant with an 11-base pair deletion in *mcrs1* gene (Supplementary Fig. S5). We crossed *mcrs1* heterozygous mutants to generate homozygous *mcrs1* mutants (Supplementary Fig. S5). As shown in Fig. 4A, homozygous *mcrs1* mutants exhibited a curved body axis at 72 hpf. The size of the brain and the eye decreased significantly at 72 and 96 hpf (Fig. 4B). To test whether the size reduction was ascribed to excessive cell death, we performed acridine orange staining, which is widely used to detect apoptotic cell death in zebrafish. We observed an increase in the number of cells stained with acridine orange in the central nervous system of homozygous *mcrs1* mutants at both 48 and 72 hpf (Supplementary Fig. S6). This suggests that cell survival in the developing central nervous system depends on mcrs1 function, although zygotic expression of mcrs1 appears to be dispensable for early organogenesis. Confirming the specificity of the observed phenotype, injection of *mcrs1* mRNA rescued excessive apoptosis in homozygous *mcrs1* mutants (Supplementary Fig. S6).

Developing zebrafish retina establishes multiple cell layers, which are clearly visible after 72 hpf. We examined if retinal lamination is affected by *mcrs1* mutantion. As shown in Fig. 4C, the retinal sections from wild-type animals at 72 hpf exhibited layered structures, whereas homozygous *mcrs1* mutants failed to form any retinal layers. Moreover, we observed a substantial increase in the number of apoptotic cells exhibiting higher-levels of active caspase 3 (Supplementary Fig. S6). Before the establishment of multiple cellular and neuritic layers, retinal progenitor cells undergo interkinetic nuclear migration, which results in mitotic cells localized to the outer (ventricular) surface. To examine interkinetic nuclear migration in *mcrs1* mutants, we next performed phospho-histone H3 (pHH3) immunofluorescence staining, which labels mitotic nuclei. In wild-type animals at 48 hpf, pHH3-positive nuclei were localized along the outer surface of the retina (Supplementary Fig. S6). In *mcrs1* mutants, pHH3-positive nuclei localized to the inner area were observed more frequently, and the number of pHH3-positive cells near the ciliary marginal zone was increased (Supplementary Fig. S6). These observations suggest that *mcrs1* mutation affects neurogenesis and interkinetic nuclear migration in the retina.

Zebrafish skin melanocytes aggregate their melanosomes in response to melatonin. The aggregation is thought to be mediated by cytoplasmic dynein^34^. To test if mcrs1 is involved in dynein function *in vivo*, we analyzed the extent of melanosome aggregation after melatonin treatment. In wild-type siblings, melanosomes were efficiently aggregated by melatonin treatment for 10 min. By contrast, melatonin treatment exerted only minor effects on melanosome movement in homozygous *mcrs1* mutants (Fig. 4D). This result suggests that mcrs1 is required for cytoplasmic dynein-dependent trafficking in zebrafish.

We next examined the formation of cilia in the olfactory placode, using immunostaining with anti-acetylated tubulin antibody as a cilium marker. Compared with wild-type siblings, there was a large reduction in the number and length of olfactory cilia in homozygous *mcrs1* mutants, demonstrating the involvement of mcrs1 in ciliogenesis (Fig. 4E). Cilia in the Kupffer’s vesicle are required for the left-right (LR) axis formation in zebrafish^35^. Our whole-mount *in situ* hybridization detecting the cardiac marker *cmlc2* showed that the direction of cardiac looping along the LR axis was unaffected by homozygous *mcrs1* mutation (Supplementary Fig. S6). Thus, it is unlikely that zygotic expression of mcrs1 is necessary for ciliogenesis in the Kupffer’s vesicle.

## Discussion

In this study, we show that MCRS1 interacts with cytoplasmic dynein and is required for the establishment of centriolar satellites. Several centrosomal and basal body proteins are present at centriolar satellites, and their incorporation into the centrosome/basal body has been shown to be influenced by centriolar satellites^24^,^29^. As expected, depletion of MCRS1 caused a reduction in the recruitment of Pericentrin to the centrosome. Moreover, we found that MCRS1 physically interacts with TTBK2, and plays a role in the recruitment of TTBK2 to the mother centriole in serum-starved RPE1 cells. Accordingly, MCRS1 knockdown in RPE1 cells affected both the removal of CP110 cap from the mother centriole and the assembly of primary cilia. Homozygous *mcrs1* mutation in Zebrafish caused a defect in olfactory ciliogenesis.

Although MCRS1 has been identified as a binding partner of NDE1, we found that MCRS1 and NDE1 play distinct roles in dynein-dependent cellular processes. First, depletion of NDE1 did not affect the localization of centriolar satellites, whereas MCRS1 is critical for dynein-dependent trafficking of centriolar satellites. Second, perinuclear localization of the Golgi apparatus is impaired in cells depleted of either NDE1 or cytoplasmic dynein. However, depletion of MCRS1 did not noticeably affect the localization and morphology of Golgi membranes. These findings suggest that MCRS1 might regulate the activity of dynein as an adaptor molecule having different cargo selectivity from NDE1. The reduction in physical interaction between dynein intermediate chain and NDE1 in cells overexpressing MCRS1 further suggests that MCRS1 and NDE1 do not act cooperatively in dynein functions. Moreover, MCRS1 and NDE1 play a positive and a negative role in the formation of primary cilia, respectively. NDE1 appears to sequester dynein light chain LC8 at the basal body, suppressing the ability of intraflagellar dynein (cytoplasmic dynein 2) to contribute to cilium formation^21^. In ciliated cells, GFP-tagged MCRS1 largely disappeared from satellites. Instead, MCRS1-GFP was enriched in cilia, suggesting a role for MCRS1 in the maintenance of primary cilia. Both cytoplasmic dynein 1 and 2 function as complexes, and share some of the accessary subunits. Thus, it needs to be tested if MCRS1 may interact and function with the cytoplasmic dynein 2 complex for retrograde intraflagellar transport, or may act to antagonize NDE1 activity.

It has been reported that zebrafish carrying a nonsense mutation in cytoplasmic dynein heavy chain (*dync1h1*) survive until larval stages, due to maternal protein stores, and die between 6–8 days after fertilization^36^. However, *dync1h1* mutant embryos already exhibit smaller eyes at 3.5 dpf, and retinal layer formation is defective^36^. Similarly, *mcrs1* mutant embryos exhibited smaller eyes and retinal lamination defect, supporting the idea that mcrs1 plays a role in dynein function *in vivo*. Moreover, *mcrs1* mutant zebrafish showed a delay in melatonin-induced melonosome aggregation, which is known to require cytoplasmic dynein^34^. Zebrafish with *dync1h1* mutation exhibit a darkened appearance due to dispersion of melonsomes in melanophores^36^. Interestingly, morpholino-mediated knockdown of several ciliopathy genes, such as *bbs4, cep290* and *rpgr*, in zebrafish also causes a delay in retrograde transport of melanosome^37^,^38^,^39^. Given that ciliopathy proteins commonly localize to centriolar satellites, one possible explanation for this phenotype is that defective centriolar satellites might sequester functional cytoplasmic dynein complexes from melanosomes. Impaired dynein function might be a major cause of extensive cell death observed in the central nervous system and retina of *mcrs1* mutant zebrafish. However, because divergent properties and functions have been attributed to MCRS1, it would be difficult to determine exact causes of the cell death.

Previously, it has been shown that MCRS1 is involved in mitotic spindle formation, and thus loss of MCRS1 causes defects in chromosome segregation^10^. MCRS1 is thought to stabilize kinetochore microtubules through inhibiting the microtubule-depolymerizing activity of MCAK, a kinesin-13 subfamily of motor protein. Mitotic defect could provide explanation for the abnormal expression of MCRS1 in various cancer tissues^4^,^6^,^40^,^41^. In mitosis, cytoplasmic dynein contributes many activities at different stages, including chromosome movements, spindle organization, spindle positioning and mitotic checkpoint silencing. Complex interactions between core subunits of dynein complexes and various binding partners may underlie multiple mitotic functions of dynein. It is possible that mitotic defects in cells expressing abnormal levels of MCRS1 might be caused not only by kinetochore-fiber destabilization but also by dysregulation of dynein activity. A recent study showed that MCRS1 is essential for the activation of mTOR complex 1 and its associated cellular functions^9^. MCRS1 appears to maintain Rheb, a regulator of mTOR, at lysosomal surface in active GTP-bound form to activate mTOR. Interestingly, dynein-dependent transport is required for the localization and activation of mTOR complex 1^42^. Thus, it is tempting to speculate that MCRS1 might mediate the link between dynein and mTOR pathway. Future studies should better clarify the involvement of dynein in many functions of MCRS1.

## Materials and Methods

### Cell culture and transfection

RPE1 and HEK293T cells were purchased from American Type Culture Collection. RPE1 cells were cultured in DMEM/F12 (Welgene) supplemented with 10% FBS and penicillin/streptomycin. HEK293T cells were cultured in DMEM (Welgene) supplemented with 10% FBS and penicillin/streptomycin. Human *MCRS1* cDNA was cloned into pEGFP-N3 (Clontech) and pCMV-Tag4 (Stratagene) plasmids. Truncated MCRS1 sequences were generated by PCR, and cloned into the pEGFP-N3 vector. Addgene provided DsRed-CENT2 (Plasmid #29523) and DYNC1I1-GFP (plasmid #37389). Human NDE1 and TTBK2 cDNAs (Open Biosystems) were cloned into pcDNA3.0 with MYC-tag and pEGFP-N3, respectively. RPE1 cells were transfected with plasmids using Lipofectamine LTXplus (Invitrogen), and analyzed 16–24 hr after transfection. For RNAi, cells were transfected with siRNAs using Lipofectamine RNAiMAX (Invitrogen), and analyzed 72 hr after transfection. Ciliogenesis in RPE1 cells was induced by serum starvation (0% FBS) for the indicated times. The following siRNA sequences were uses: MCRS1 #1, AGCUCAAGGACAUGCGAGA; MCRS1 #2, GGCAUGAGCUCUCCGGACU; PCM1 #1, GGCUUUAACUAAUUAUGGA; PCM1 #2, UCAGCUUCGUGAUUCUCAG; NDE1 #1, GGACCCAGCUCAAGUUUAA; NDE1 #2, GCGCAGACCAAAGCCAUUA; NDE1 #3, GCUGAAGCCUGUUCUUGGU; NDE1 #4, GCAGCACUCUGAAGGCUAC; DYNC1l1 #1, GGAAGGCACUGUUGAGUUA; DYNC1l1 #2, GGAAAUUCGUGCUAACAGA; DYNC1l1 #3, CAAGGGAAGUAGUGUCCUA; DYNC1l1 #4, CGGGAGACGUCAAUAACUU. Unless specifically stated, experiments were performed using pools of the siRNAs targeting each gene.

### Immunoblotting and immunoprecipitation

Cells were scraped and lysed by sonication in RIPA lysis buffer (20 mM HEPES, pH 7.5, 150 mM NaCl, 1% NP-40, 0.25% sodium deoxycholate and 10% glycerol) containing Protease Inhibitor Cocktail (Sigma). Protein concentrations were determined by the BCA assay. Samples were separated by SDS PAGE, transferred onto Hybond-C extra nitrocellulose membrane (GE Health Sciences) and immunoblotted with the indicated antibodies. For co-immunoprecipitation assay, HEK293T cells were lysed with Nonidet P-40 lysis buffer 16 hr after plasmid DNA transfection, and cell lysates were incubated with anti-FLAG M2 affinity gel (Sigma) or GFP-Trap (Chromotek) overnight at 4 °C. Immunoprecipitates were boiled with 2 x sample buffer (125 mM Tris-HCl, pH 6.8, 4% SDS, 20% glycerol and 0.004% bromphenol blue), and the same volume of samples were loaded for immunoblot analysis.

### Immunofluorescence

Cells were fixed in methanol for 4 min at −20 °C or in 4% PFA for 8 min at room temperature (RT). In case of PFA fixation, cells were permeabilized with 0.1% Triton X-100 (Sigma-Aldrich). After blocking with 5% FBS for 10 min, cells were incubated with primary antibodies at RT for 1 hr. Primary antibodies were detected by 1 hr incubation with Alexa Fluor 488-, 594- or 647-conjugated secondary antibodies (Life Technologies). Cells were mounted with mounting solution (75% glycerol, 20 nM Tris, and 0.02% NaN_3_) containing 0.25 μg/ml of DAPI (Sigma-Aldrich). This study used the following antibodies: rabbit anti-MCRS1 (Proteintech), mouse anti-MCRS1 (sigma), mouse anti-α-Tubulin (Sigma), rabbit anti-PCM1 (Cell signaling), rabbit anti-CEP290 (Abcam), mouse anti-Pericentrin (Abcam), mouse anti-γ-tubulin (Abcam), rabbit anti-γ-tubulin (Abcam), mouse anti-Giantin (Abcam), mouse anti-polyglumamylated tubulin (AdipoGen), rabbit anti-ARL13B (Proteintech), rabbit anti-TTBK2 (Proteintech), rabbit anti-CP110 (Proteintech), mouse anti-acetylated tubulin (Sigma), mouse anti-FLAG (Sigma), rabbit anti-FLAG (Sigma), mouse anti-MYC (Cell Signaling), rabbit anti-MYC (Abcam), and rabbit anti-GFP (Abcam).

### Microscopy and image analysis

Images of fixed or live cells were generated using 20 × (0.4 NA) or 60 × (1.4 NA) Plan Apo objective lens on an Olympus IX70 microscope equipped for optical sectioning microscopy (Deltavision; Applied Precision). For quantification of cellular phenotypes (CEP290 localization, Golgi organization and ciliogenesis), randomly selected 6–8 fields for each experiment were imaged using 20 × objected lens. To quantify CP110 cap removal and immunostaining intensities of Pericentrin and TTBK2, localization of the centrosome was determined by staining with anti-γ-tubulin antibody, and more than 100 cells imaged using 60 × objective lens were randomly selected for each experiment. Integrated fluorescence intensity in the centrosomal area was measured using ImageJ software after background subtraction. PhotoShop CS6 (Adobe Systems) was used to construct final figures. The differences between experimental values were considered significant when P-value was <0.05 in an unpaired Student’s t-test using GraphPad Prism 5.0.

### Generation of zebrafish mutants

TALEN plasmids targeting the exon 4 of zebrafish *mcrs1* gene were designed and constructed by ToolGen: left TALEN, TTGAGCCAATCAGATGCCCT; right TALEN, ATGTCCAGCGACAAGAAAAA. TALEN plasmids were linearized with PvuII (Takara Bio) and purified with gel extraction kit (Elpis Biotech). Then, *mcrs1* TALEN mRNAs were generated using the mMESSAGE mMACHINE T7 Transcription kit (Ambion) according to the manufacturer’s instructions. Zebrafish embryos at the 1-cell stage were microinjected with the TALEN mRNAs. After the activity of the TALEN was confirmed, the injected larvae were raised to adulthood (F0). F0 fish were out-crossed with wild type to produce stable KO line (F1). For genotyping of F1 KO mutant fish, PCR and T7E1 assay were performed. The following primers were used for PCR: mcrs1-F0-FP, GTCTGCTGTGGGTGCAGGATCGT; mcrs1-F0-RP, TGGAGCCAGCACCTAAGCT; mcrs1-F1-FP, TGGTTGAAAGTAGCCTCGCA; mcrs1-F1-RP, TTCTTGTCGCTGGACATG. The study protocol was approved by Institutional Review Board of Chungnam National University and all experiments were carried out in accordance with the approved guidelines.

### Whole-mount *in situ* hybridization

Zebrafish embryos were staged by morphological features and fixed in 4% PFA. Embryos were treated with 0.00 3% phenylthiourea (PTU) to inhibit pigment formation. Anti-sense RNA probes for *mcrs1* and *cmlc2* was synthesized from cDNA templates using DIG-RNA labeling kit (Roche). Whole-mount *in situ* hybridization was performed as described previously^43^.

### Acridine orange staining

For detection of apoptotic cells, zebrafish embryos were incubated with 10 ug/ml acridine orange (Sigma) for 30 min in the dark, and washed several times in egg water (60 ug/ml sea salts, Sigma).

### Zebrafish histology and immunofluorescence

For histological analysis, zebrafish embryos at 48 hpf and 72 hpf were fixed overnight in 4% PFA at 4 °C, dehydrated with a graded ethanol, processed in xylene and embedded in paraffin. Cross section (6 μm) were stained with hematoxylin and eosin using a standard protocol^44^. For whole mount immunofluorescence staining, zebrafish embryos at 48 hpf were fixed overnight in 4% PFA and dehydrated with methanol. Zebrafish embryos were permeabilized in acetone for 7 min at −20 °C and washed in water, followed by several washes in PBST. After blocking for 30 min in 2% horse serum, zebrafish embryos were incubated with active caspase-3 antibody (BD Biosciences) or p-histone H3 antibody (Millipore) at 4 °C overnight. On the next day zebrafish embryos were incubated with Alexa Fluor 568-conjugated secondary antibody (Invitrogen). Cryosection was performed as described previously^45^.

### Melanosome aggregation analysis

Melatonin (Sigma) was dissolved in DMSO and diluted in egg water. The zebrafish embryos were embedded in 3% methyl cellulose and treated with 10 mM melatonin. After treatment, aggregation of melanosomes was monitored for 30 min.

## Additional Information

**How to cite this article**: Lee, S.-H. *et al*. MCRS1 associates with cytoplasmic dynein and mediates pericentrosomal material recruitment. *Sci. Rep.* **6**, 27284; doi: 10.1038/srep27284 (2016).

## Supplementary Material

Supplementary Information
