# Supplementary material for: MCRS1 associates with cytoplasmic dynein and mediates pericentrosomal material recruitment
Source: Sci Rep. 2016 Jun 6;6:27284. doi: 10.1038/srep27284 (PMC4893664; doi:10.1038/srep27284)
Supplement: Supplementary Information [file srep27284-s1.doc]

**Supplement information**

**MCRS1 associates with cytoplasmic dynein and mediates pericentrosomal material recruitment**

Si-Hyung Lee^1^, Mi-Sun Lee^2^, Tae-Ik Choi^2^, Hyowon Hong^1^, Jun-Young Seo^3^, Cheol-Hee Kim^2*^, and Joon Kim^1*^

^1^ Graduate School of Medical Science and Engineering, KAIST, Daejeon 34141, Korea.

^2^ Department of Biology, Chungnam National University, Daejeon 34134, Korea.

^3^ Severance Biomedical Science Institute, Brain Korea 21 PLUS Project for Medical Science, Yonsei University College of Medicine, Seoul 03722, Korea.

**
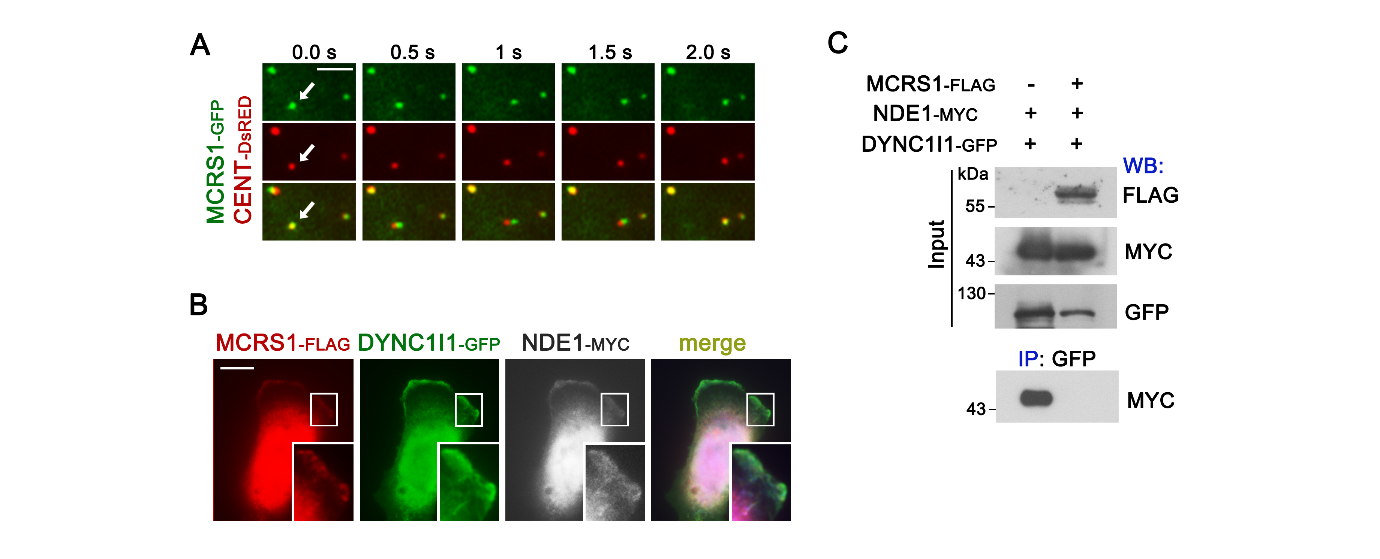
**

Fig. S1. Interaction between MCRS1 and cytoplasmic dynein. (A) Time-lapse imaging of a live RPE1 cell expressing MCRS1-GFP and Centrin-DsRed for 2 sec. (B) Immunofluorescence images of a RPE1 cell stained with anti-FLAG and anti-MYC antibodies after transfection with MCRS1-FLAG, DYNC1l1-GFP and NDE1-myc plasmids. Insets are magnified view of lamellipodia. (C) HEK293T cells were transfected with the indicated plasmids for 16 hr, and then cell lysates were immunoprecipitated with anti-GFP antibody conjugated with agarose beads. The resulting precipitates and input lysates were immunoblotted with the indicated antibodies. Scale bars represent 2 μm (A) and 10 μm (B).

**
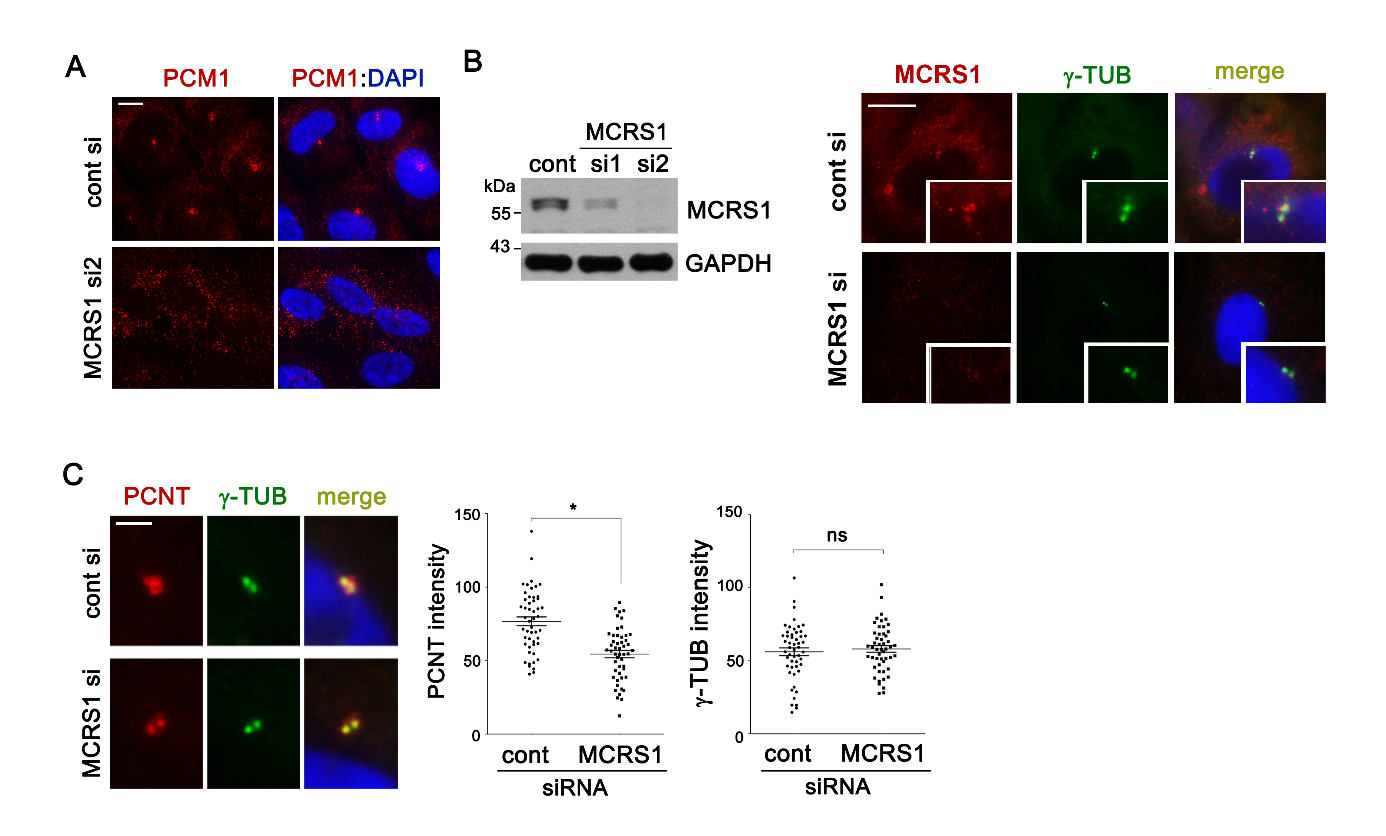
**

Fig. S2. Depletion of MCRS1 and its effect on pericentrosomal material recruitment. (A) Immunofluorescence images showing PCM1 distribution in RPE1 cells after transfection with MCRS1 siRNA 2. (B) Western blot and immunofluorescence analyses demonstrating efficient knockdown of MCRS1 in RPE1 cells transfected with MCRS1 siRNAs. (C) Immunofluorescence images showing Pericentrin (PCNT) and γ-Tubulin after MCRS1 knockdown. The scatter plots show quantification of immunofluorescence intensities of Pericentrin or γ-Tubulin in the centrosomal area. Error bars represent SEM (more than 100 cells were examined for each group; *P < 0.05, t test; ns, not significant). Scale bar represent 10 μm (A) and 2 μm (C).

**
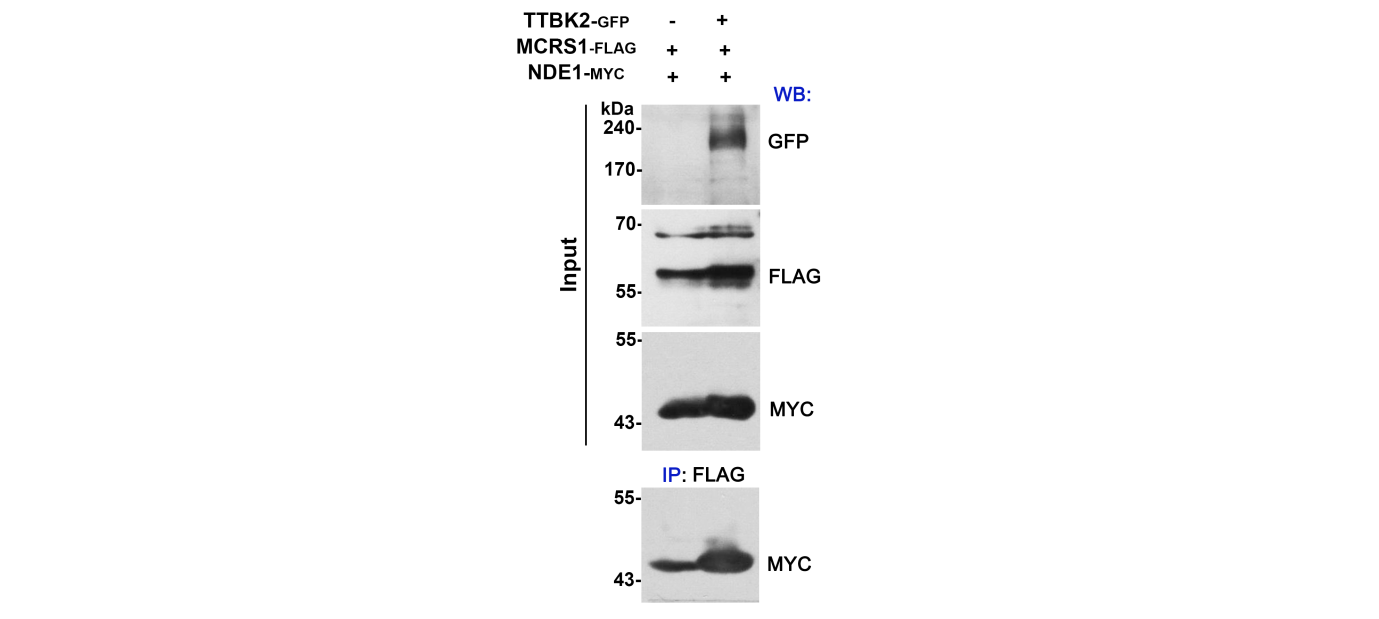
**

Fig. S3. TTBK2 overexpression causes an increase in the physical interaction between MCRS1 and NDE1. HEK293T cells were transfected with the indicated plasmids for 16 hr, and cell lysates were immunoprecipitated with anti-FLAG antibody conjugated with agarose beads. The resulting precipitates and input lysates were immunoblotted with the indicated antibodies.

**
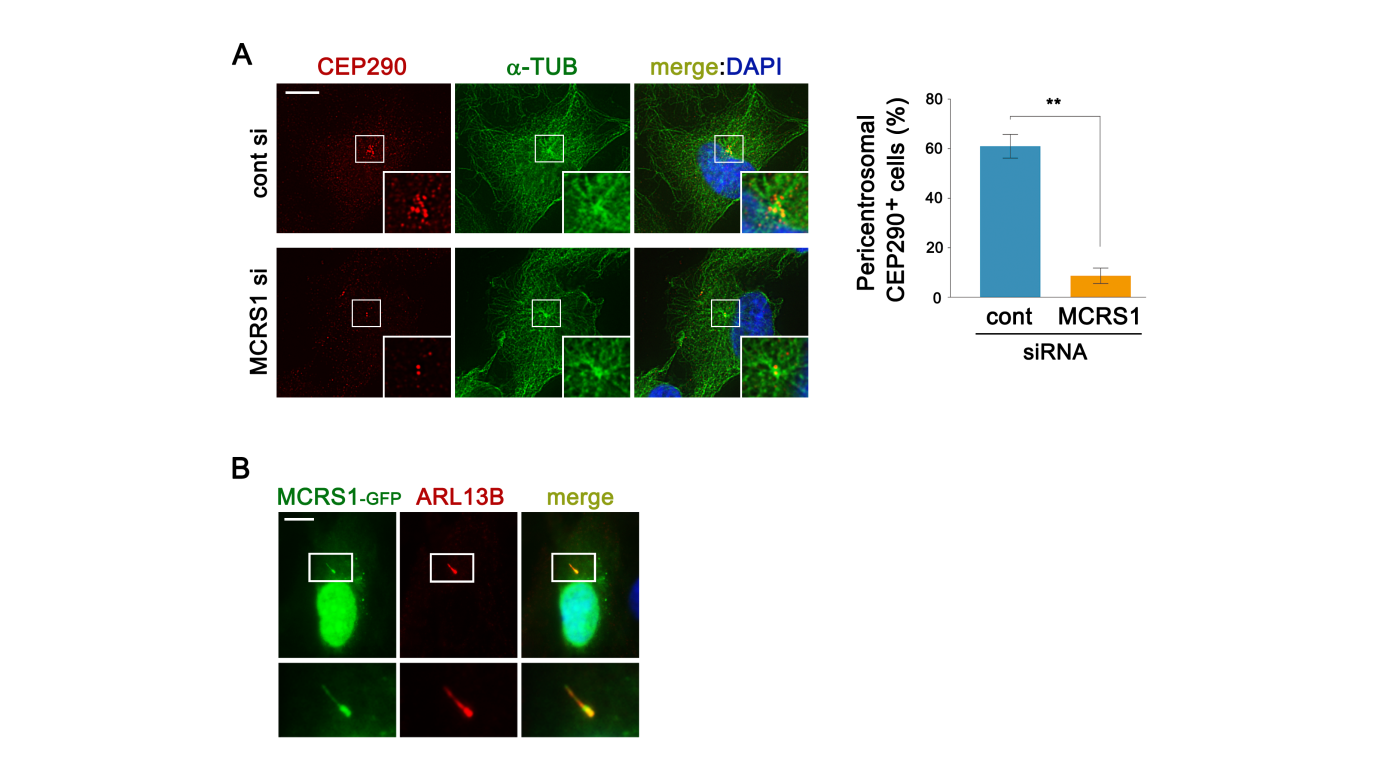
**

Fig. S4. MCRS1 is required for proper distribution of CEP290. (A) Immunofluorescence images showing microtubules and the localization of CEP290 after MCRS1 knockdown. The graph shows quantification of cells exhibiting pericentrosomal accumulation of CEP290. Error bars represent SEM (n=3 independent experiments; **P < 0.01, t test). (B) Localization of MCRS1-GFP in a ciliated RPE1 cell. Cells were transfected with MCRS1-GFP plasmid, and ciliogenesis was induced by serum starvation for 16 hr. Cilia were stained with anti-ARL13b antibody. Scale bars represent 10 μm.

**
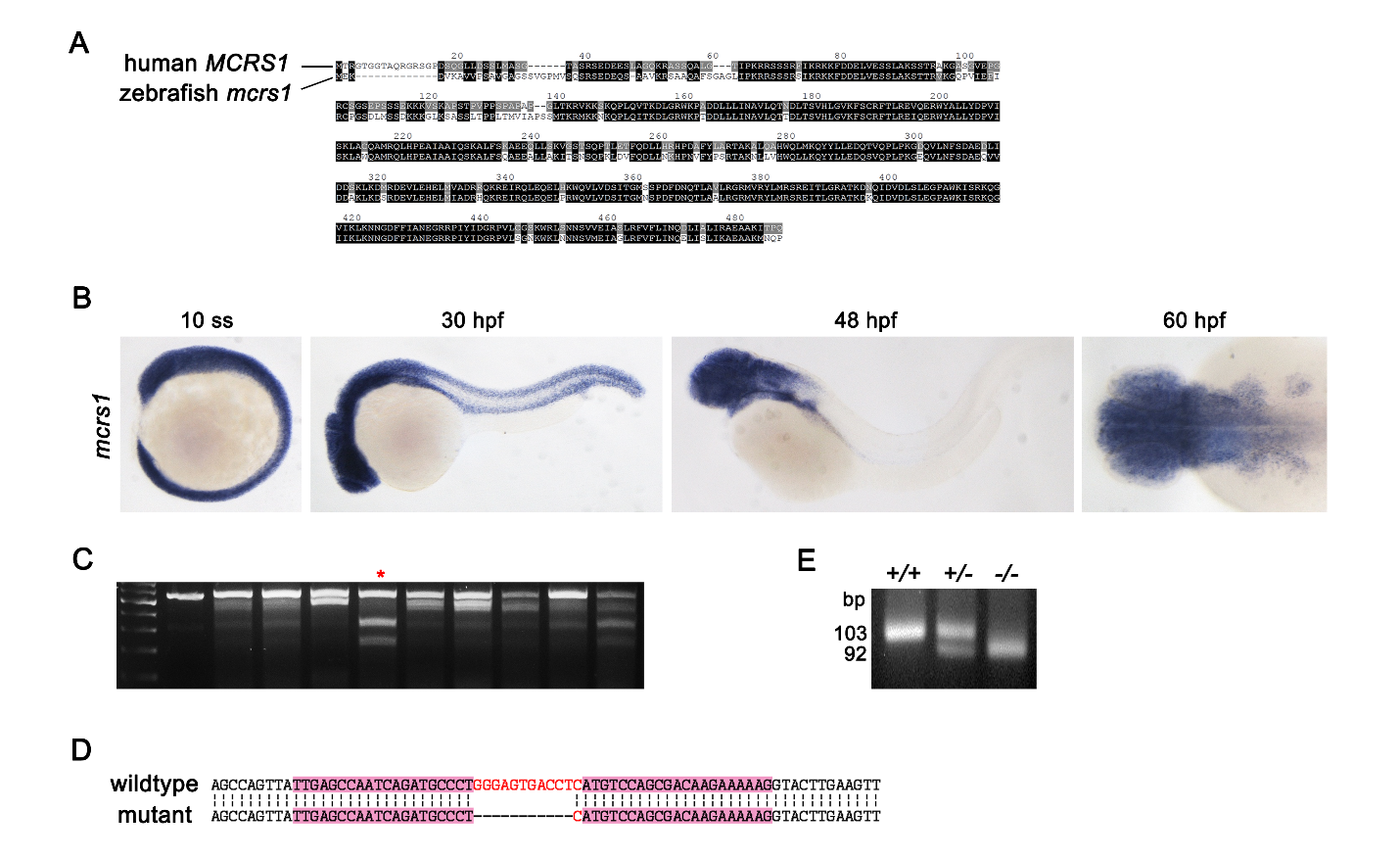
**

Fig S5. TALEN-mediated knockout of *mcrs1* in zebrafish. (A) Alignment of human and zebrafish MCRS1 protein sequence (ClustaIX program). (B) Expression of *mcrs1* mRNA during embryonic development was examined by whole-mount in situ hybridization (ss, somite stage; hpf, hours after fertilization). (C) T7E1 cleavage assay for the identification of zebrafish carrying a deletion in *mcrs1* gene. Cleaved bands in lane 5 indicates *mcrs1* het mutation. (D) Deletion of 11 base pairs in *mcrs1* gene exon 4 was confirmed by DNA sequencing. (E) Genotyping of *mcrs1* mutants using PCR.

**
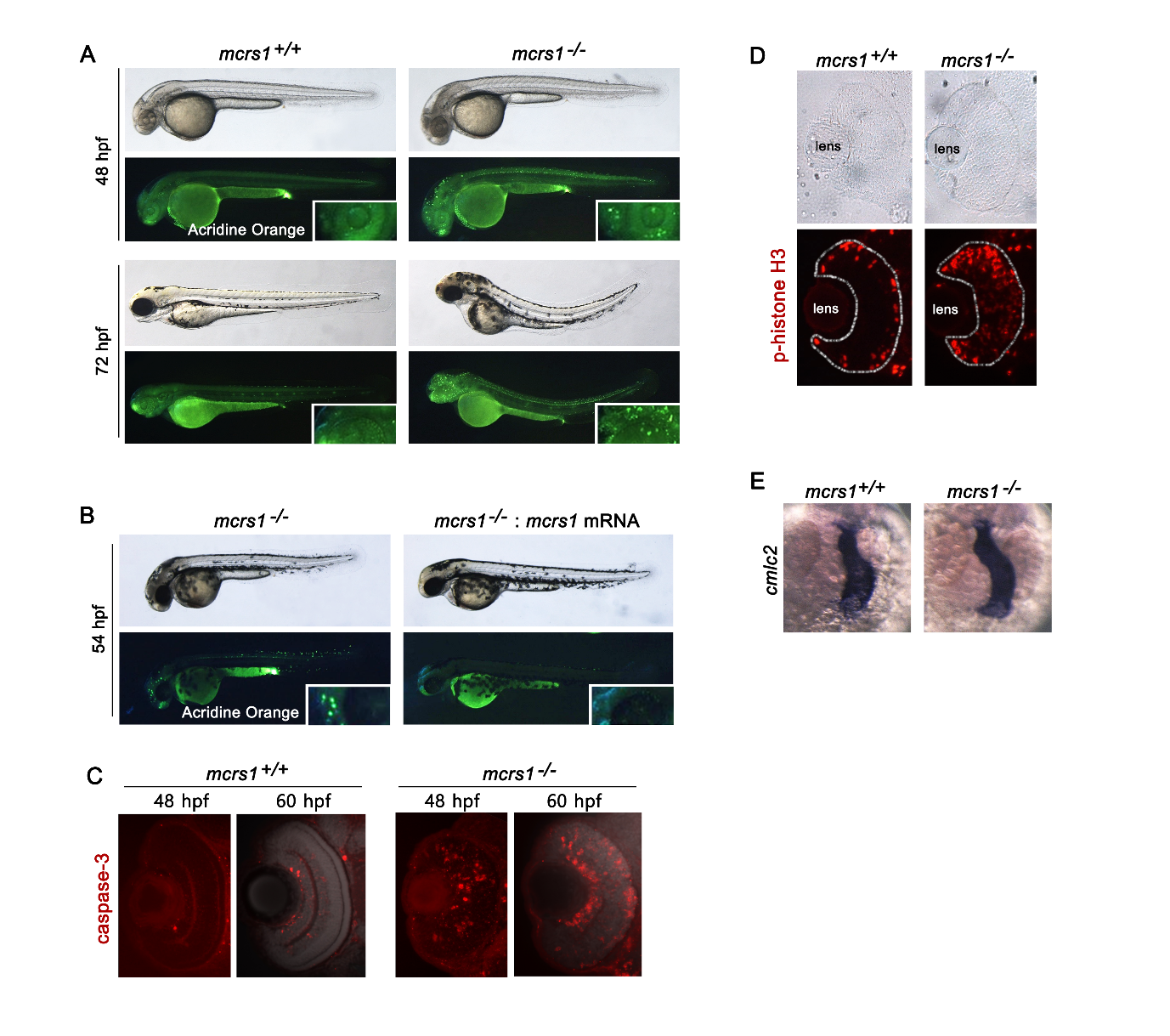
**

Fig S6. Phenotype of homozygous mcrs1 mutant zebrafish. (A) Homozygous mcrs1 mutants showed increased apoptotic cell death in the central nervous system. Apoptotic cells were detected by acridine orange staining at 48 and 72 hpf. Insets are magnified view of the head. (B) Injection of mcrs1 mRNA rescued excessive apoptotic cell death in mcrs1 mutant zebrafish. (C) The retinal sections from embryos at 48 and 60 hpf were stained with anti-active caspase3 antibody. (D) The retinal sections from embryos at 48 hpf were stained with anti-phospho-histone H3 antibody. (E) Whole-mount in situ hybridization of cmlc2. The establishment of left/right body axis is normal in homozygous mcrs1 mutants.
